# Organic Medicinal and Aromatic Plants: Consumption Profile of a Portuguese Consumer Sample

**DOI:** 10.3390/foods12224145

**Published:** 2023-11-16

**Authors:** Ana Mendes, André Oliveira, Jorge Lameiras, Pedro Mendes-Moreira, Goreti Botelho

**Affiliations:** 1 Regional Directorate of Agriculture and Fisheries of the Center (DRAPC), Coimbra Delegation, Av. Fernão de Magalhães, 465 RC, 3000-177 Coimbra, Portugal; ana.mendes@drapc.gov.pt; 2 Polytechnic Institute of Coimbra, Coimbra Agriculture School, Bencanta, 3045-601 Coimbra, Portugal; bioandre2018@gmail.com (A.O.); pmm@esac.pt (P.M.-M.); 3 Shared Care Resources Unit, Health Centers Group of Baixo Mondego, Regional Health Administration of Center, 3000-075 Coimbra, Portugal; jmmoreira@arscentro.min-saude.pt; 4 Research Centre for Natural Resources Environment and Society (CERNAS), Polytechnic Institute of Coimbra, Bencanta, 3045-601 Coimbra, Portugal

**Keywords:** medicinal and aromatic plants, organic farming, consumption habits, health, questionnaire

## Abstract

The production and consumption of organic products have been increasing in Portugal, as well as in the European Union as a whole. The main objective of this work is to understand the consumption habits of organic medicinal and aromatic plants (OMAPs) among Portuguese adults. An online questionnaire was distributed using social networks, resulting in the collection and statistical analysis of 300 responses. Of the participants who reported consuming OMAPs, 44.3% showed a daily consumption pattern. The most frequently mentioned OMAPs for fresh consumption were parsley (*Petrosselinum crispum* L., 92%), garlic (*Allium sativum* L., 84.1%), and coriander (*Coriandrum sativum* L., 78.1%). The most commonly mentioned OMAP for consumption as dried plants were oregano (*Origanum vulgare* L., 74.6%), lemon balm (*Melissa officinalis* L., 49.2%), and lemon verbena (*Aloysia citrodora* L., 46.8%). The main reasons cited for using OMAPs were their benefits to health (58.7% of participants), benefits to the environment (33.2%), and reduced salt consumption (29.5%). Among these, the main health benefits mentioned included anti-inflammatory properties (45.0%), prevention of cardiovascular diseases (41.6%), and prevention of high cholesterol (39.9%). Furthermore, 82.5% of respondents considered themselves sufficiently, well, or excellently informed about the nutritional properties of OMAPs. This research initiates a discussion about whether profiling OMAP consumption habits can serve as a valuable tool for promoting organic farming in Portugal, increasing OMAP production and consumption, and strengthening the connection between these products and potential positive human health effects.

## 1. Introduction

Aromatic plants are characterized by great botanical diversity, morphological characteristics (as different sizes—herbaceous, semi-woody, and woody), and physiological characteristics (as different life cycles), but they have a common feature of aromatic and/or medicinal properties [1].

The European Union (EU) defines medicinal and aromatic plants (MAPs) as plants that are used first and foremost owing to their medicinal or aromatic properties in pharmacy or perfumery [2]. There are plants used mostly as aromatic plants, as they are important sources of essential oils used in different industrial areas to give fragrance and taste. Medicinal plants have a pharmacological action and are used in the prevention, relief, or cure of diseases. These two properties (aromatic and medicinal) may or may not exist in the same plant, their degree of importance being highly variable between species [3,4,5]. In fact, medicinal plants are commonly used in the prevention and treatment of specific diseases and illnesses and are generally considered to have a beneficial role in health [6].

Several ethnobotanical and ethnopharmacological studies indicate properties that are attributed to MAP and the direct and indirect benefits that their use can bring to humans [7,8,9].

Along with their importance in the food industry, MAPs and/or their derivatives also play a relevant role in the cosmetic industry, being valued for this reason [2,10]. In addition to contributing to the physical, mental, and social well-being of people in the community, these plants can play an important role in the balance of ecosystems, especially in agricultural ecosystems [11,12,13].

Under good agricultural practices followed for the cultivation of the MAPs, organic cultivation is of utmost importance. Organic manures not only help in improving soil health but also help in better growth of plants and do not have any synthetic phytopharmaceutical residual effect on plant produce [14].

Due to its climate characteristics, the Mediterranean region stands out for being a place where the most different and distinct aromatic plants can be found, such as rosemary, oregano, coriander, sage, mint, and thyme [15].

One of the principles of the Mediterranean Diet is the presence of aromatic plants in the daily diet, which gives the amalgamation of flavors and aromas that are traditionally recognized in this dietary pattern [16].

The Portuguese Flora is very important for its richness in aromatic [17] and medicinal plants. In reality, of the approximately 3800 species that make up the vegetation cover of the mainland, Azores and Madeira, around 500 are aromatic and/or medicinal, and some of them may constitute an alternative for sustainable agricultural systems or for the profitability of marginal land for agriculture [18].

The new generations, the interest in healthy lifestyles, and even the growing existence of people affected by diseases such as diabetes, hypertension, cardiovascular diseases, and cancer encourage the consumption of organic products [19]. In addition, despite the growing interest in OMAPs on the part of consumers, we could not find previous information on preferences regarding the places of purchase, their presentation (packaged or in bulk), and their status (fresh, dry/dehydrated, or frozen), and the preferred mode of consumption (raw, cooked, infusions, etc.).

The main objective of this study was to contribute to understanding the consumption habits of OMAPs using a convenience sample of 300 Portuguese adults. To achieve this goal, several issues were addressed, including the reasons leading people to consume OMAPs, the acquisition sources, the most valued agricultural production modes, the most consumed fresh and dried products, the frequency of their use, the perception regarding their main possible benefits for human health, and the sources of information used.

## 2. Materials and Methods

The survey was accomplished using a convenience sample based on the availability of access to a digital platform and the willingness to answer the questionnaire. A snowball methodology via email and social media contacts was used to scatter invitations to participate. Although convenience samples may have some disadvantages, they are very useful for exploratory research [20,21].

The study included individuals 18 years of age and over. Participants under 18 were excluded because they were under legal age. Since the questionnaire was applied via computational means, it was not feasible to obtain authorization from the legal guardians.

All the participants were volunteers. Responses were anonymously collected. While developing the questionnaire and collecting the data, all ethical issues were respected following the Declaration of Helsinki. Each participant could only access the questionnaire after being informed that no personal identification would be collected, after agreeing to participate, and after expressing informed consent. The data was kept strictly confidential so that none of the responses could ever be linked to the participant. The study was submitted for evaluation and approved by the Ethics Committee of the Polytechnic Institute of Coimbra (reference Nº 52_CEIPC/2022, 23 February 2022).

A Web questionnaire was prepared using the Google Forms tool (Google Inc., Mountain View, CA, USA), with 30 questions aggregated into three different sections. There were obtained 300 valid answers, being 74% women and 26% men. Descriptive statistical data analysis was performed in Microsoft^®^ Excel^®^ for Microsoft 365 MSO (version 2301 Build 16. 0. 16026. 20002) 64-bit. The chi-square test was calculated in SPSS (IBM SPSS Statistics for Windows, version 26.0., Armonk, NY, USA) following the statistical procedures described by Howell [22].

The questionnaire is structured in three parts: (1) Fourteen questions directed to sociodemographic and socioeconomic characterization of the sample, with a set of questions related to the place of acquisition and presentation of the MAPs and a partition question, regarding the concern with their production methods; from that point, the ongoing participants were requested to answer to (2) Ten questions aimed at identifying the most consumed OMAPs, their habits, and frequency of consumption and method of preparation and confection; and (3) Four questions related to the perceived main benefits of OMAPs and knowledge about their nutritional and chemical properties, as well as the level of quality of information and knowledge and the acquisition channels.

The questions directed to the sociodemographic and socioeconomic characterization of the sample were shared with another recently published study about the consumption of organic fruits and vegetables [23].

## 3. Results

The first set of questions was directed to make the sociodemographic and socioeconomic characterization of the sample.

Regarding gender, there was a predominance of female respondents (73.7%). About the distribution of individuals in the sample by age classes, the age groups between 35–44 years old and between 45–54 years old are represented equally (26.7%), followed by the age group 25–44 years old (20.3%). The participants with an age higher or equal to 65 years account for just 2.3%. The use of electronic forms as a means of collecting responses may explain the lower prevalence of the older age group (≥65 years). Observing academic qualifications, 74% of respondents have higher education qualifications. Concerning monthly net income classes, most respondents (40.5%) have a monthly net income varying between €991 and €1950, while 3.3% of the respondents reported an amount of less than €635, and another 3.3% of the sample have values greater than €4851 (Table 1).

Most of the participants in this survey were concerned about the way MAPs are produced since about 2/3 of the respondents (201 respondents) admitted to being sensitive to this criterion, while one-third (99 participants) expressed no concern about this issue. The participants in this study who stated that they were not concerned or careful with the way in which the products they consumed were produced ended their participation in this survey here.

From those participants in the survey continuing the questionnaire, 62.2% of the respondents to the survey who were concerned about the MAPs production method stated that their preference was for Organic Farming, and around 50.2% of the respondents showed a preference for products from Conventional Farming. The Integrated production method registered 10% of the answers, and the Biodynamic and Permaculture production methods registered 0.5%.

The results obtained allow identifying the female gender as being mainly responsible for the acquisition of OMAP, with 58.3%. The male gender is only responsible for the purchase in 8.7% of the cases, and 33.0% of the respondents stated that they shop with another member of the household.

The person responsible for purchasing the OMAP is mostly in the age groups between 35 and 54 years old, comprising 65% of the sample.

Most of the participants have a family comprising only two persons (54.7%). Families of three are mentioned by 20% of participants, and 19% live alone.

Considering the region of Portugal, 64.7% are from the Centro region. This difference from other regions might be just circumstantial, resulting from the social network contacts.

Of the total number of responses obtained to this question, 49.3% of the participants revealed that it is in large/medium-sized stores that they usually purchase OMAP, while 28% of them prefer to purchase them at markets/producer fairs and 26.3% of participants in the survey admitted buying these products in traditional shopping areas. Direct purchase of OMAP from the producer only occurs in 25.7% of respondents.

OMAPs are available in several presentations since their use is also diversified. Most respondents (63.7%) prefer purchasing fresh and in bulk, while 40% opt for packaged fresh products. About 22% buy OMAPs in the dehydrated state in bulk, while 8.7% buy them in the packaged dehydrated state. Only 7.3% opt for the purchase in frozen mode.

The identification of the OMAPs most chosen by the participants is part of the set of questions in the second part of this study.

**Figure 1:**
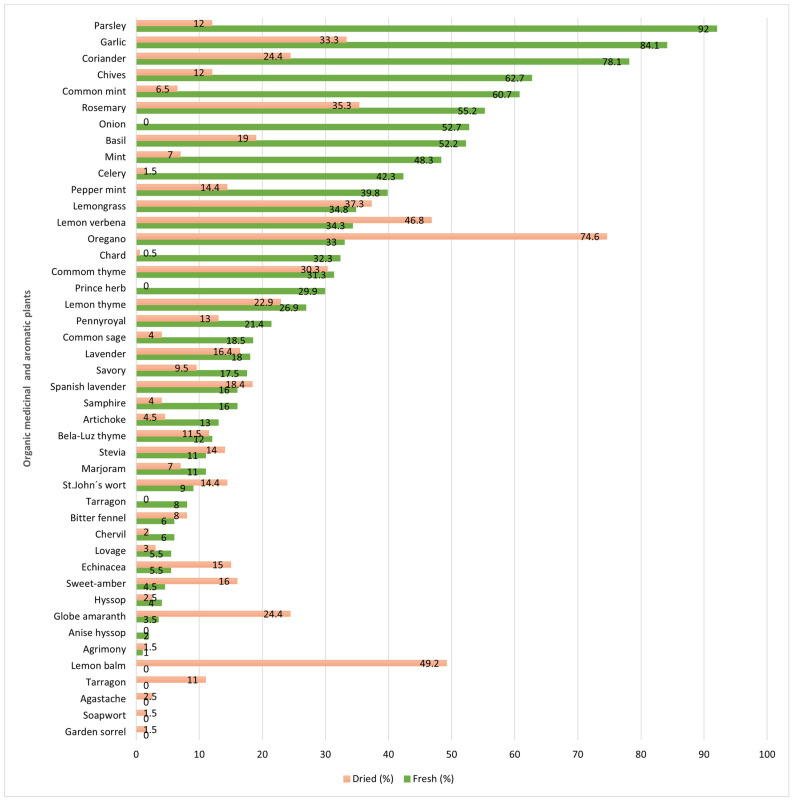
The OMAPs are mostly consumed as fresh or dried products.

The OMAPs most consumed as a fresh product were parsley (*Petrosselinum crispum* L.) 92%, garlic (*Allium sativum* L.) 84.1%, coriander (*Coriandrum sativum* L.) 78.1%, chives (*Allium schoenoprasum* L.) 62.7%, and mint (*Mentha spicata* L.) 60.7%. Among dried products, the standout OMAPs for consumption were oregano (*Origanum vulgare* L.) 74.6%, lemon balm (*Melissa officinalis* L.) 49.2%, lemon verbena (*Aloysia citrodor* L.) 46.8%, lemongrass (*Cymbopogon citratus* L.) 37.3%, and rosemary (*Rosmarinus officinalis* L.) 35.3% (Figure 1).

Another set of questions in the study is intended to find out the consumption habits of the survey respondents, as well as the years and frequency of OMAP consumption in the context of the household.

The participants in this study, when asked about other organic products they usually consume, expressed interest in the following products: legumes (mentioned by 66.7% of participants), seeds (56.7%), and cereals (52.2%). Organic dairy products and meat products had a number of responses corresponding to 42.8% and 41.8%, respectively. Considering the 8.4% of respondents that mentioned some other organic products on the market, there is a wide range of products, but eggs are the most chosen among them by consumers (28.6% of respondents), followed by cocoa/chocolate (14, 3% of responses).

Most of the participants in this survey (44.3% of the answers), have a daily consumption of OMAP. From the remaining respondents, 15.9% referred to consuming OMAP 5 to 6 times a week, and 14.4% claimed to consume 3 to 4 times a week.

The use of OMAPs is quite versatile, and they can be consumed in numerous ways. According to the data obtained, OMAPs are mostly consumed raw (66.7%). Addition during roasting was the next preferred option (mentioned by 65.1% of respondents), followed by addition during the cooking process, which was mentioned by 57.2% of respondents.

OMAPs can be consumed throughout a meal, from the beginning as appetizers to the end as dessert. According to the data obtained, OMAPs are mostly used in the main dish, as indicated by 74.1% of participants. Soups come next at 34.8%, but they are also consumed indiscriminately throughout the meal, a preference that obtained 28.9% of the possible options.

When it comes to using OMAPs in beverages, infusions are the most popular choice, with 77.1% of respondents favoring them. Smoothies follow as the next option, chosen by 26.3% of participants, and juices are the third most chosen option, corresponding to 22.9%. Detox drinks, as a more recent alternative, are preferred by 10.4% of respondents.

The use of OMAPs provides consumers with the freedom to use and consume them in various ways, depending on individual preferences. It appears that most consumers (55.7%) prefer to create their own mixtures. Using OMAPs individually is the next choice, selected by 25.9%, while only 17% of respondents opt for recommended blends. In cooking and food preparation, 55.8% of women and 60% of men prefer to use their own mixtures. Individual use is made by 27.9% of women and 20% of men. Using recommended blends is chosen by 16.2% of women and 20% of men. The difference between men and women in the distribution of OMAP use in the cooking/preparation of food was not statistically significant (χ^2^(2) = 1.231, *p* = 0.540).

A third set of questions related to the perceived main benefits of OMAPs and knowledge about their nutritional and chemical properties, as well as the level of quality of information and knowledge obtained from different acquisition channels.

Undoubtedly, the primary reasons for consuming OMAPs are their benefits to health (58.7% of respondents). Concern for the environment comes next (33.2% of responses), followed by the desire to reduce salt consumption (29.5% of responses). Sensory reasons, such as enhancing the taste of meals, were indicated by 24.5% of respondents (Table 2).

As participants were allowed to select multiple options, each benefit was individually analyzed in terms of gender differences using the Chi-square test applied to a 2X2 contingency table.

Statistically significant differences were found in the option “Benefits to health” (χ^2^(1) = 4.365, *p* = 0.037), which was chosen by 48.7% of men and 62.3% of women, and in the option “Reduced salt consumption” (χ^2^(1) = 6.810, *p* = 0.009), which was chosen by 17.9% of men and 33.6% of women.

Focusing specifically on health-related reasons, Table 3 highlights the most relevant health benefits associated with the consumption of OMAPs. Notably, anti-inflammatory properties were recognized as relevant by 45.0% of respondents. Additionally, the prevention of cardiovascular diseases and the prevention of high cholesterol were indicated by 41.6% and 39.9% of participants, respectively. The prevention of gastrointestinal diseases and hypertension prevention also ranked high, with each being an important reason for 36.9% of the sample.

The role of OMAPs in preventing obesity was recognized by 24.4% of men and 36,8% of women, and this difference was statistically significant (χ^2^(1) = 4.009, *p* = 0.045). There was also a statistically significant difference (χ^2^(1) = 4.475, *p* = 0.034) for “Anti-aging properties”, being mentioned by 19.2% of men and 31.8% of women.

**Figure 2:**
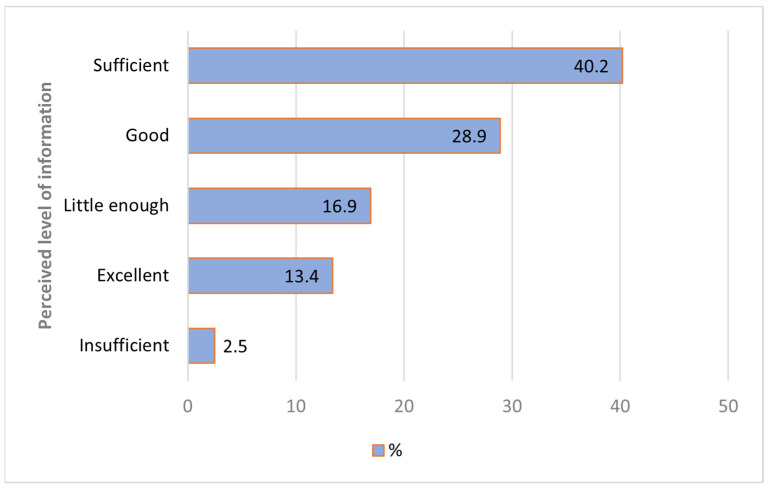
Perceived level of information about OMAP.

In Figure 2, we assess the level of information that consumers have regarding the nutritional, chemical, and other properties of OMAPs. The option with the highest number of responses was a “sufficient” level with 40.2% of respondents. The levels “good” and “excellent” received 28.9% and 13.4% of responses, respectively. The option “little enough” was chosen by 16.9% of respondents. This indicates that more than 95% of participants feel they have at least some or even a high level of information, which empowers them to make informed choices in acquiring and using OMAPs. The “insufficient” level of knowledge was mentioned by only 2.5% of the participants.

Statistically significant differences for respondents considering the perceived level of knowledge about OMAPs and the main health-related reasons for consuming OMAPs were not found.

**Figure 3:**
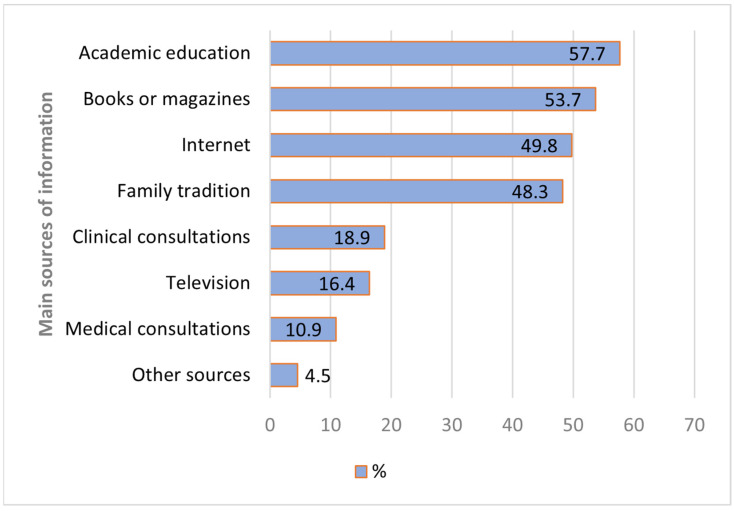
Main sources of information about OMAPs.

The academic path emerged as the primary source of knowledge regarding the benefits of consuming OMAPs, gathering the highest number of responses at 57.7%. Following closely, books and magazines were cited as a means of knowledge with 53.7%. The internet, accessible to a vast majority of the population, accounted for approximately 49.8% of responses. Family tradition also plays a significant role in the transmission of knowledge related to OMAP consumption, as this option was selected by 48.3% of the respondents (Figure 3).

The perceived knowledge is lower (the only statistically significant difference) for respondents who mentioned the internet (U = 4032.5, *p* = 0.015) or television (U = 1902.5, *p* = 0.011) as sources of information about OMAPs.

## 4. Discussion

When considering the general demographics of the respondents, this study aligns with other studies that show a predominance of females. However, it is not entirely clear whether this signifies an effective predominance among consumers of MAPs or if it relates to a greater role in household food management and food choices [24,25,26,27] or bias for answers in the general population rather than household concerns. In contrast, there are only a few studies with a predominance of male respondents [26,28].

In this study, the age groups 35–44 years old and 45–54 years old are equally represented (26.7%), followed by the age group of 25–44 years old (20.3%). This differs from several other studies in which the age groups of 25–34 (or under 35) and 35–44 (or under 45) years old are predominant [25,26,28]. Another study shows a more homogenous distribution among adults [27]. Furthermore, the present study found a higher percentage of respondents with higher education qualifications, as a few other studies have also noted [26,27]. In contrast, several other studies found a predominance of respondents with high school or secondary education or even less [24,25,26,28].

Medicinal and aromatic plants are the sources of various bioactive components used as the basic materials for various pharmaceuticals, food additives, flavors, and industrially important biochemicals. These bioactive components have been used by mankind for treating various diseases for a long time. Many of these bioactive components have very complex structures, making them hard to synthesize economically in the laboratory, and thus, plants remain their only source of production [14].

It is expected that the demand for MAPs will increase in both developed and developing countries in the future. This growth can be attributed to the increasing global population and a growing awareness of the economic and health benefits of natural products, particularly when they are perceived as safe and effective alternatives to synthetic medicines [25].

The most up-to-date information available from the Portuguese General Directorate for Agriculture and Rural Development indicates that in 2012, around 1759 hectares were explored by 249 OMAP producers. The most recent numbers are from 2017, indicating that around 855 ha are explored by 377 OMAP producers. It appears that although the area has decreased between 2012 and 2017, the number of producers has increased from 249 to 377 [29].

According to the study of the Aromatic, Medicinal, and Spice Plants sector in Portugal, MAP production is carried out predominantly in organic mode, representing 89% of the total [4].

Production of MAPs provides an opportunity for small-scale farming, even at the family level, to supply local markets and families. It also contributes to the preservation of traditional agricultural landscapes and generates opportunities for the management of natural resources and the preservation of the genetic heritage of agricultural products [30].

The belief or at least the expectation of consumers regarding unprocessed MAPs can be a valuable aspect in marketing for OMAPs. Simultaneously, there is a pressing need to enhance widespread knowledge about the benefits, safe dosage, and potential side effects of pharmaceutical usage of MAPs. These conditions create an opportunity for investment in educational training for natural gatherers, farm producers, and sellers. Moreover, discussions are needed on potential protection policies and rules for natural gathering to ensure the sustainability of these plants in the face of increasing demand [28].

Implementing training programs and providing farmers with seeds can make a significant contribution to ensuring the natural sustainability of MAPs while also generating economic income for farmers and rural communities. When it comes to herbalists and sellers, fostering professional competency and responsibility can be achieved using specialized training, alongside public supervision, to safeguard public health [24].

The increase in consumer information and interest in OMAPs means an economic potential to enhance production, but attractive prices for the producer and market channels are important to add value to production, counteracting the uncertainty of net income generated from traditional seasonal crops and market uncertainty. In fact, from a food perspective, considering both medicinal and aromatic plants, under the climatic shifts and low productivity of traditional field crops, there is a risk of pushing farmers toward medicinal crops for profit maximization [31].

Concerns about organic farming include procedures to control weeds in crop fields. MAPS may be a viable alternative because some of their bioactive compounds may act as bioherbicides [32] and green pesticides [33], and so MAPs have the potential to perform as ecologically sustainable resources for organic agri-systems, safeguarding the sustainability of farming alongside minimizing toxic risk for humans and ecosystems.

The organic production of MAPs can be developed on lands with no other agricultural use or on those used for agricultural production but without the application of synthetic chemicals. Intercropping in the organic cultivation of medicinal and aromatic plants seems to be a simple and very efficient method to reduce the occurrence of pests and diseases since the use of pesticides is not allowed in the organic production system. Plant cultivation in this system contributes to increased biodiversity, better utilization of natural resources, higher yields of many plants, and reduced abundance of weeds and pests [34].

Sustaining plant species and conservation of genetic resources have to be a priority in human concerns about biodiversity, especially facing the risks associated with climate change worldwide [8].

MAPs are exposed to the effects of climate change, like all other plants. Even though the effects of climate change on medicinal plants, in particular, have not been well studied and are not fully understood, it is quite possible to think that changes in plant structure and composition may occur, affecting the geographical distribution, survival, or the genetic integrity of some MAPs. Climate change’s impact may have a tremendous possible effect on MAPs, particularly significant due to their value within traditional systems of medicine and as economically useful plants [35].

Despite the widespread importance attributed to Medicinal and Aromatic Plants (MAPs), even in studies conducted in countries directly located or near the Mediterranean basin, there are variations in the most used MAPs. These differences are likely more associated with cultural traditions than with climate and agricultural potential [24,25,27,28]. Cultural factors play a significant role in shaping preferences for botanical species, their intended uses, and methods of usage [28].

A study conducted in 2012 in Portugal showed that the production sector of MAP is centered on the segment of organic MAP for drying [36], and this condition has been reaffirmed in the most recent results of projects on the sector in Portugal [4].

In fact, literature mentions herbal infusions or decoctions (tisanes) as one of the most economical and practical uses of plants as MAP, considering their interest provided by the presence of active biomolecules present as secondary metabolites in those vegetal structures. Herbal tisanes are produced purely from a single plant or as a combination of more than one herb with different active ingredients. Although most herbal tisanes are consumed for their pleasant taste, some herbal tisanes are used for health protection due to medicinal or preventive effects on health, namely antioxidant effects [5].

The specific content of nutrients and bioactive molecules and the presence of non-volatile compounds (such as phenolics, contributing to the specific sensory properties) also means that the phytochemical profile of plant species containing specific and complex mixtures of bioactive molecules provides numerous opportunities for the development of new categories of food and beverages. Given consumers’ increasing concerns about their health, OMAPs open new challenges in technological, ecological, and economic aspects to enable more sustainable production processes and the enrichment of functional products already present on the market or for the development of new ones tailored to target consumers. This also presents commercial challenges for achieving satisfactory and desirable nutritional and sensory quality, as well as assured stability and shelf-life of the products [37].

Aromatic plants, also known as “herbs and spices”, have been widely used for bactericidal, fungicidal, virucidal, antiparasitic, insecticidal, medicinal, cosmetic, agronomic, and sanitary applications since ancient times [38,39]. Moreover, the value-added effects of aromatic plant derivatives permit us to understand their potential benefits to health. For instance, due to their unique characteristics, such as anti-inflammatory, antioxidant, antimicrobial, and antiseptic properties, they should be of great importance in the food, pharmaceutical, and cosmetic industries. After classifying them as Generally Recognized as Safe (GRAS), they could be used as food ingredients to improve food quality and develop novel functional foods [40]. The antimicrobial and antioxidant activities of the major natural bioactive compounds of some aromatic plants used in the food industry are well summarized by these authors.

In this study, apart from one exception, a specific association was not found between the health reasons mentioned for the use of OMAPs and self-perceived knowledge. Considering a potential correlation between self-perceived knowledge and information sources, it appears that the internet and television are two insufficient sources to instill confidence in consumers regarding their knowledge. These information sources may require additional investment as they present an opportunity to reach a more informed consumer base for OMAPs.

The Mediterranean Diet is recognized as a feeding pattern with multiple benefits on health and nutrition status [41,42]. It also represents a whole way of living embracing diverse, sustainable values, with a positive impact on health, society, economy, and the environment [43,44]. Beyond that, it is a good representative of a sustainable food system because it’s based on an ancestral food production system respecting biodiversity, providing a balanced nutritional composition of food, the safety of food based on natural fertilizers, and a low environmental footprint, the positive impact on the local economy, and food security, and a communal sense and identity [43,45]. But two specific features are quite interesting to mention: the valorization of aromatic plants, especially as alternatives to salt used in cooking procedures, and the valorization of the cultural and familial context sharing knowledge about food preparation and cooking.

The prevalence of inadequate sodium intake is high in the Portuguese population, with 76.4% of the population exceeding the maximum tolerated value. This prevalence is higher among adults (79.7%) and adolescents (74.7%) [46]. Incorporating herbs and spices into reduced-salt food items is a feasible strategy for achieving a significant reduction in salt content without sacrificing the sensory pleasure associated with food [47].

It should be considered that not all users of MAPs have the same attitude towards those products. In fact, they may use them out of curiosity or choose them with a more intentional rationale based on knowledge. For this, consumption seems to be correlated with the safety and efficacy perception of natural products, healthcare concerns, and environmental protection, also exhibiting a growing interest in environmentally certified MAP products [26]. It is also important to perceive that purchasing behavior is not only determined by sensibility to environmental sustainability, food safety, and health concerns, but there are also effects from income, education/information level, and responsiveness to advertising. It cannot be neglected that less informed individuals may adopt a skeptical attitude about consumption because of possible side effects [25], and low income may impair access to MAPs, especially if people do not have minimal conditions for home production.

It is important to draw attention to some of the current study’s limitations. The main drawback of this study is the size of the sample. In fact, it was expected at the beginning of the study to reach a large number of participants, but due to the channel chosen to disseminate the survey, we possibly encountered many subjects who were resistant to participate in an online survey and, for this reason, did not participate in it. Another identified limitation of the study is the absence of more detailed information regarding the health condition of organic food consumers and how this ultimately influences food preferences and practices. It raises a challenge to deepen the health approach in future research. Despite its limitations, this research is essential for initiating a discussion about whether profiling the consumption habits of OMAPs can serve as a valuable tool for promoting organic farming in Portugal and boosting OMAP consumption rates among the newer generations.

Evidence from this study and others suggests that sociodemographic factors, such as gender, age, education, and income, generally play a significant role in determining consumption behavior regarding Medicinal and Aromatic Plants (MAPs) as a whole. However, more refined studies reveal consumer segmentation by combining these sociodemographic factors with other conditions, including origin (natural gathering, conventional farming, organic farming), sources of information about products, place of purchase, product presentation (fresh in bulk, packaged, dehydrated, etc.), organoleptic characteristics, price or brand factors, safety perception (from natural origin, from farming production, adverse reactions and effects from improper use), traceability from origin to market, ecological certification and environment and biodiversity protection, main interest for use (medicinal or aromatic), perception of phototherapeutic efficacy, scientifically proven benefits [25,26].

The primary purpose of these profiles is to gain a deeper understanding of the consumption behavior of MAPs. They serve various useful purposes, including supporting public policies from production to the market, providing robust information for action, developing training and information resources for producers and sellers, planning educational initiatives to empower consumers in making safe and efficient choices with MAPs, informing health professionals about preventive and therapeutic opportunities using MAPs, adjusting production and market offerings to meet consumer needs, and refining marketing strategies [25,26].

The rising interest in herbs and their economic applications holds great significance in the context of a shift toward greener economies and lifestyles [48]. Furthermore, given the growing importance of environmental concerns for younger generations, this presents a valuable opportunity to promote awareness and literacy about OMAPs. It allows for the sharing of a cultural treasure, including the accumulated knowledge, information, and plant materials passed down from generation to generation, enriching our understanding of these plants worldwide.

## 5. Conclusions

A high proportion of respondents (201 respondents) from 300 participants in the survey were concerned about the agricultural mode of production (organic farming, conventional farming, permaculture, or other) in which MAPs are produced, while one-third (99 participants) expressed no concern about this topic. The consumption of OMAPs on a daily basis was referred by 44.3% of the 201 participants.

According to our study, the most frequently mentioned OMAPs for fresh consumption were parsley (*Petrosselinum crispum* L., 92%), garlic (*Allium sativum* L., 84.1%), and coriander (*Coriandrum sativum* L., 78.1%). The most stated OMAP for consumption as dried plants were oregano (*Origanum vulgare* L., 74.6%), lemon balm (*Melissa officinalis* L., 49.2%), and lemon verbena (*Aloysia citrodora* L., 46.8%). The main reasons cited for using OMAPs were benefits to health (58.7% of participants), followed by benefits to the environment (33.2%), and reduced salt consumption (29.5%). Among these, the main health benefits mentioned included anti-inflammatory properties (45.0%), prevention of cardiovascular diseases (41.6%), and prevention of high cholesterol (39.9%). More than 95% of participants stated that they have at least some or even a high level of information important enough to make informed choices in the acquisition and use of OMAP.

Organic foods, including OMAPs, have been increasingly valued for human health and well-being and for their importance in sustainable farming, protecting the environment, and providing community welfare. There is a recognition of a broad range of benefits for humans, nature, and society, rewarding the investment of individuals and communities.

To the best of our knowledge, this study is the first that directly provides detailed information on OMAP consumption profiles among Portuguese adults. Consumers appear to prefer consuming medicinal and aromatic plants from organic farming over conventional farming, and they recognize the benefits of these plants in promoting health and preventing disease.

## Figures and Tables

**Table 1:** Summary of academic qualifications and net income of participants (adapted with permission from [23]. Copyright 2023, De Gruyter).

Education Level	Total (*n* = 300)		Female (*n* = 221)		Male (*n* = 79)	
	*n*	%	*n*	%	*n*	%
Elementary school (4th year to 9th year)	19	7.0	9	4.07	10	12.66
Secondary education or Professional course	57	19	40	18.09	17	21.52
Higher education (Bachelor, Master, Doctorate)	222	74	170	76.92	52	65.83
Monthly net income						
Less than 635 €	10	3.29	8	3.57	2	2.5
Between 991 €–1950 €	122	40.45	103	46.43	19	24.05
More than 4851 €	10	3.29	3	1.34	7	8.86

*n*—number of participants.

**Table 2:** Main reasons for consuming OMAPs.

	Total		Female		Male			
	*n*	%	*n*	%	*n*	%	χ^2^	*p*
Benefits to health	175	58.7	137	62.3	38	48.7	4.365	0.037 *
Benefits to environment	99	33.2	76	34.5	23	29.5	0.664	0.415
Reduced sugar consumption	59	19.8	47	21.4	12	15.4	1.296	0.255
Reduced salt consumption	88	29.5	74	33.6	14	17.9	6.810	0.009 **
Sensory reasons	73	24.5	56	25.5	17	21.8	0.417	0.518
Fashion	2	0.7	1	0.5	1	1.3		

* *p* < 0.05; ** *p* < 0.01. *n*—number of participants.

**Table 3:** Main health-related reasons for consuming OMAPs.

	Total		Female		Male			
	*n*	%	*n*	%	*n*	%	χ^2^	*p*
Prevention of high cholesterol	119	39.9	94	42.7	25	32.1	2.736	0.098
Prevention of diabetes	92	30.9	70	31.8	22	28.2	0.352	0.553
Prevention of cardiovascular diseases	124	41.6	97	44.1	27	34.6	2.128	0.145
Prevention of gastrointestinal diseases	110	36.9	86	39.1	24	30.8	1.712	0.191
Prevention of hypertension	110	36.9	84	38.2	26	33.3	0.581	0.446
Prevention of obesity	100	33.6	81	36.8	19	24.4	4.009	0.045 *
Anti-aging properties	85	28.5	70	31.8	15	19.2	4.475	0.034 *
Anti-inflammatory properties	134	45.0	105	47.7	29	37.2	2.589	0.108

* *p* < 0.05. *n*—number of participants.

## Data Availability

The original database is available from the corresponding author upon reasonable request.
